# Replication of Leaf Surface Structures for Light Harvesting

**DOI:** 10.1038/srep14281

**Published:** 2015-09-18

**Authors:** Zhongjia Huang, Sai Yang, Hui Zhang, Meng Zhang, Wei Cao

**Affiliations:** 1 School of Mechanical and Automotive Engineering, Anhui Polytechnic University, Wuhu 241000, China; 2 Soochow University-Western University Centre for Synchrotron Radiation Research, Institute of Functional Nano and Soft Material (FUNSOM) and Collaborative Innovation Center of Suzhou Nano Science & Technology, Soochow University, Suzhou 215123, China; 3 Department of Physics, East China University of Science and Technology, Shanghai 200237, China; 4 Research Center for Molecular Materials, University of Oulu, P.O. Box 3000, FIN-90014, Finland

## Abstract

As one of the most important hosts of natural light harvesting, foliage normally has complicated surface structures to capture solar radiances. Bio-mimicking leaf surface structures can provide novel designs of covers in photovoltaic systems. In this article, we reported on replicating leaf surface structures on poly-(methyl methacrylate) polymers to prompt harvesting efficiencies. Prepared via a double transfer process, the polymers were found to have high optical transparencies and transmission hazes, with both values exceeding 80% in some species. Benefiting from optical properties and wrinkled surfaces, the biomimetic polymers brought up to 17% gains to photovoltaic efficiencies. Through Monte-Carlo simulations of light transport, ultrahigh haze values and low reflections were attributed to lightwave guidance schemes lead by the nano- and micro-morphologies which are inherited from master leaves. Thus, leaf surface bio-mimicking can be considered as a strategic direction to design covers of light harvesting systems.

In front of fossil fuel depletion^1^ and global warming^2^, novel development in renewable energy production is one of the most urgent and crucial issues at the present century. As the most abundant but also a clean source within human’s disposal, solar energy can be converted and stored naturally in plants or in artificial light harvesting systems. Materials to protect solar cells are supposed to have high transparency and transmission haze rate to scatter light and subsequently increase photoabsorptions in reactive centers^3^. Elaborated efforts have been dedicated to introducing periodical nano- or micro- structures in the aim of manipulating incident light propagations^4^,^5^,^6^,^7^,^8^,^9^,^10^. Functional surfaces in 2D were further extended to wrinkled structures in 3D. It has been shown such quasi-periodical structures with an additional dimension were able to enhance light-harvesting performance in photovoltaics (PV)^11^. Furthermore, successful incorporations of wrinkled textures in capacitors^12^, piezoelectrics^13^ and solar cells^14^ have resulted in mechanically robust thin-film energy generating and storage devices. However, types of wrinkles and grains are rather tremendous compared to regular or crystalized material shapes. Setting up strategies of searching for proper texture structures becomes a new challenge in functional material sciences.

Within all light harvesting systems, the green plants play a crucial rule in converting sunlight into chemical energy. Photosynthesis happening on foliage is the principle producer of both oxygen and organic matters on earth^15^. After million years of evolution, leaves typically have optimized surface structures on upper epidermises to adapt themselves to different environments. Micro- and nano- structures with different shapes are distributed on them^16^. Surface structures of a plant leaf can actively promote photosynthesis and influence on the capture of solar radiances^17^. Additionally, leaves of different plants vary in photosynthetic abilities, efficiencies and surface structures, even though they are growing in similar natural environments. Such abundant natural prototypes offer a library of wrinkle and surface styles for artificial light harvesting covers.

Inspired by leaf surface structures and their functions in light harvesting, we replicated leaf morphologies on to poly-(methyl methacrylate) polymers in the aim of lifting light harvesting efficiencies. We studied optical properties of the leaf-mimicking poly-(methyl methacrylate) (LM-PMMAs) and changes of photovoltaic efficiencies after folding the biomimetic polymers onto the solar cells. High values of more than 80% were found in optical transparency and haze rates, denoting a key trade-off between light transmission and transmission haze. Due to the optical properties and rich textured 3D surfaces, the LM-PMMAs substantially increased light harvesting efficiencies. We further studied surface morphologies of the polymer structures, and employed them as subjects in Monte-Carlo simulations of photon transport to reveal origins of the optical properties.

## Results

### Refinement of a double transfer process

**Figure 1:**
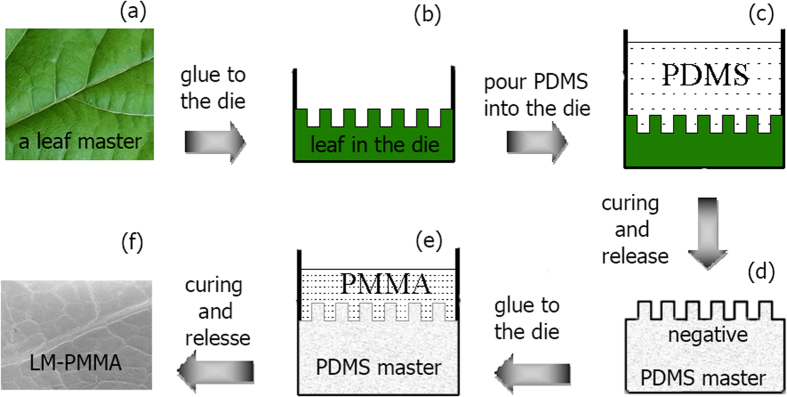
Schematic flow charts of the two-phase fabrication process. **(a)** A fresh leaf was taken as a master module. **(b)** The leaf was stuck in the die. **(c)** Polydimethylsiloxane was deposited on the leaf to get negative structures of the master. **(d)** The flexible negative mold was covered with an anti-sticking layer. **(e)** Viscous PMMA was poured onto the negative mold. The PMMA was then polymerized, degased and annealed. (**f**) After polymerization and annealing, the detached PMMA mimicked the master leaf surface structures. The photo of (**a**) was taken by Zhongjia Huang.

Plant foliage and especially the wax cuticles are softer and more fragile compared to cover materials in the artificial light harvesting systems. To realize a good surface mimicking, tender process at the replication and hard solidification at the finishing are required. Here we refined the engineering of a double transfer fabrication process^18^. A schematic flow chart is shown in Fig. 1. Starting from a clean leaf in Fig. 1 (a), the leaf was stuck to the bottom of the die used for the master structure in Fig. 1(b), and non-viscous polydimethylsiloxane (PDMS) was deposited on the leaf master to fabricate a negative mold in Fig. 1(c). After cured and released from the master, the flexible negative mold was coated with an anti-sticking layer as shown in Fig. 1(d). The methyl methacrylate (MMA) was pre-polymerized with peroxidation benzoin formyl (BPO) to obtain a low-viscous grout, and then quenched at room temperature. The partially cured viscous PMMA was subsequently poured on the negative mold (Fig. 1e). The mold and PMMA were then moved into a vacuum oven in order to remove possible gas bubbles in the system. Temperatures and ambiences were carefully optimized during the polymerization, degasing and annealing steps. The mold was warmed at 40 °C ~ 50 °C for 10 ~ 12h. After most of the pre-polymer on the mold was solidified, the mold was annealed at 100 °C and kept at this temperature for another 2h. The annealing was also carried out in vacuum to prevent oxidizations or carbonizations of the polymer in air. After the PMMA was fully cured and detached from the mold, a leaf-mimicking PMMA was obtained as shown Fig. 1(f), which presented a detailed replica of the surface morphology of the master structure in Fig. 1(a).

In total, mature leaves of 32 plant species ranging from aquatic plants to terrestrial plants were mimicked. The plant names are listed in the first row of Table 1. The double transfer process allows making LM-PMMAs of different thicknesses from 70 μm to few millimeters. To ensure methodical reliability and repeatability, at least 3 films were made per plant, while in each process a fresh leave was employed as the master. For some species, series of LM-PMMAs were synthesized with varied thicknesses from 100 μm to above 1 mm. To study optical property changes due to leaf development, 4 LM-PMMAs were also engineered with their masters of young leaves from 4 plants of corn, lotus, Photinia serrulata, and Ilex chinensis Sims. Leaves were flat, except for the young lotus leaves. Early leaves were not mimicked due to the severe leaf rolling, and rather small sizes which don’t suit most experiments in the present study. To make the optical parameters comparable when varying species, we selected 3 films per plant, with each film thicknesses around 0.60(5) mm. Their thicknesses for optical measurements were included in Table 1 after the plant names. The films have two sides, textured sides mimicking leaf surfaces, and flat ones from the polymer intrinsic shape.

### Light transparency and transmission haze rates

Results of light transparencies and transmission haze rates of the white light were tabulated in Table 1. Standard deviations in the parentheses were generated from experimental values obtained from 15 measurements: 5 measurements per an LM-PMMA, 3 films per plant. Data were organized in a descending order with haze rates. The plant names of master leaves were also listed. Hereafter, we name biomimetic leaves as LM-PMMA@PLM, where PLM stands for a plant name. Due to the transparent polymer foils of the PMMA, all the polymers have high transparencies more than 80%, except for LM-PMMA@sugarcane and @Ficus elastica. The transmission haze rates are in general, much higher than the value of plane PMMA. Clear differences are found among the haze rates of LM-PMMAs, with their values ranging from 48.9% to 87.6%. It is important to note that both high rates of more than 80% are available in LM-PMMA transparencies and hazes. This can be seen from the first 6 rows of Table 1 left (except for LM-PMMA@sugarcane), where the haze rate peaks at 87.6% of the LM-PMMA@vicaryi. Thickness impacts on optical properties of the biomimetic polymers were studied in LM-PMMA@corn. The transparencies and haze rates were depicted in the Supplementary Information. Only a slightly decrease of transparency was found, while the haze was almost unchanged within statistic error despite of the increase of the polymer thickness. Optical properties were illustrated by the results listed in Supplementary Information for the LM-PMMA films with young leaves as their masters. Similar to Table 1, Table S1 in the Supplementary Information shows the thicknesses, transparencies and transmission haze rates of LM-PMMAs made from young leaves. No obvious differences were found between optical properties of the mature and young leaves’ replications of the corn, Photinia serrulata, and Ilex chinensis Sims. However, optical haze from young lotus leaf replicas gains 10%.

**Figure 2:**
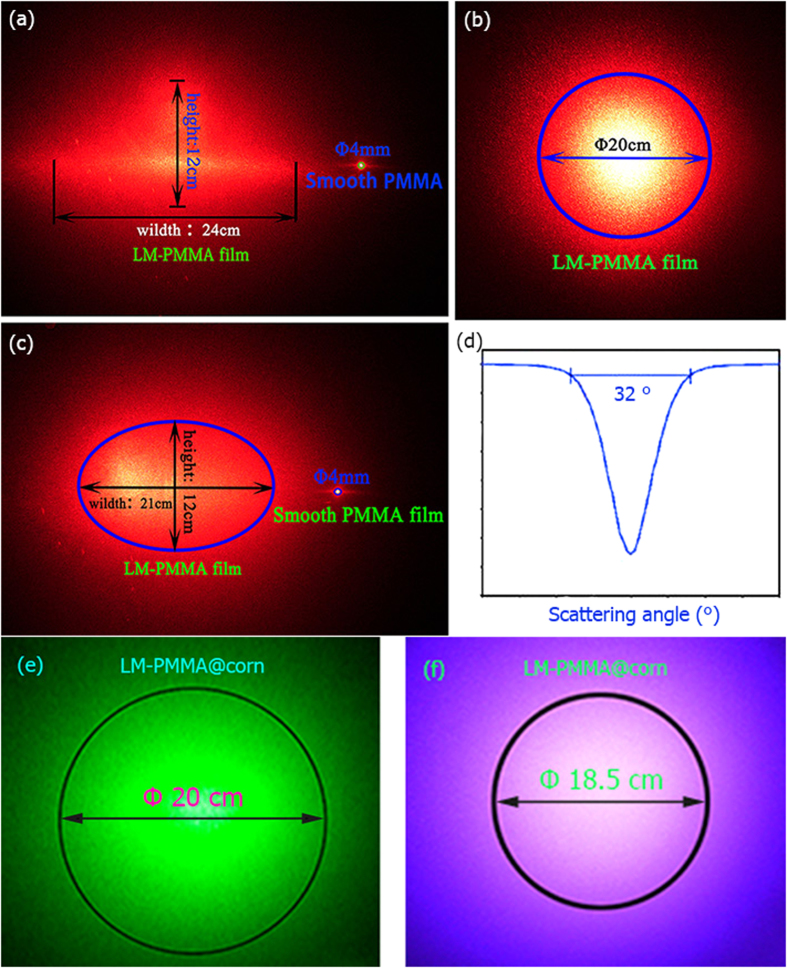
Visualizations of transmission haze for mimicked corn leaf. **(a**–**c)** show the light scattering effect of LM-PMMA@corn when a laser with a diameter of 0.3 cm passing though different places of the polymer. **(d)** Scattering angular distribution was counted with an arbitrary y-axis unit for LM-PMMA@corn, where a maximum scattering angle of 32° is found. **(e,f)** show the scattered lights of a green laser and a blue laser. Photos within the figure are in true color directly taken from a digital camera.

The transparency and transmission haze are visualized through light scattering experiments under the incident shining of a red color laser with a wavelength 650 nm and a beam diameter 0.3 cm. Laser beam passed through three different positions of the LM-PMMA@corn and illuminated large areas but with different shapes. The scattered beams were photographed and shown in Fig. 2(a–c). In contrast to a 0.4 cm spot after laser passing through the smooth PMMA, the illuminated areas are rather large due to forward scatterings of the laser. Most scattered beams are in circles (Fig. 2b), with some in ellipse (Fig. 2c), and rhombus (Fig. 2a). The laser beam was scattered to rather large spots. By counting 80% of the scattered intensity, beam area was magnified several thousand times after 32 cm of travel path. From Fig. 2(d), a large scattering angle of 32^o^ was found after the transparent polymer. This value is competitive to an angle of 34^o^ given by light interactions with the latest nano-structured paper^19^. Variations in light distributions are most probably yielded from surface micro-morphology distributions on the polymer. Light scattering visualizations were extended in another two colors of green and blue, within the visible light region. The monochromatic green (wavelength 532 nm) and blue (wavelength 405 nm) lasers were employed as incident sources, and the same LM-PMMA@corn as the target. The scattered circles were depicted in Fig. 2(e,f) for quantitative comparisons. The green circle is similar in size as the red circle in Fig. 2(b), while the blue one obviously smaller.

### Photovoltaic efficiency

**Figure 3:**
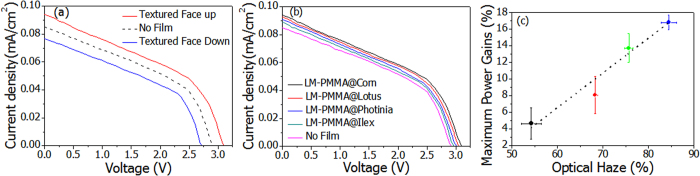
Results of photovoltaic tests. **(a)** I-V curves of LM-PMMA@corn with the mimicking film textures facing up, facing down (film reversed), and without the film were depicted in solid red, solid blue, and dashed black lines, respectively. **(b)** Four I-V curves measured by covering LM-PMMA of @Ilex chinensis Sims, @Photinia serrulata, @Lotus, LM-PMMA@corn were compared to the curve without any coverage. **(c)** Maximum gains of solar cell powers after covering artificial polymer increase with transmission haze rate provided similar film optical transparencies.

Polymers with high transparency and transmission haze will have potential applications in light harvesting systems^20^,^21^. Furthermore, the wrinkled structures substantially increase surface areas, which give larger interaction regions to capture solar radiances^22^. Photovoltaic tests were carried out with and without attaching the LM-PMMAs onto photocells of different qualities. Parameters of PV cells were tabulated in the Supplementary Information. The I-V curves were measured via laminating replicated corn surface facing up, facing down (film reversed), and without the film onto a used PV unit with a shunt resistance to imitate daily usages. Curves were plotted in solid red, solid blue, and dashed lines in Fig. 3(a), respectively. A clear increase of electrical power was detected comparing the red and black curves. This increases start from short-circuit current to the open-circuit voltage. The max power point was shifted from 2.15 eV in bare cell case to 2.27 eV in the one covered with the polymer. Consequentially, the maximum power gained 17%. It is also interesting to note that reversing the film will decrease light harvesting efficiencies, as shown by the blue curve where relatively low currents were generated at the same voltages. To examine efficiency dependence on mimicked species, we carried out the same I-V measurements with another three polymers of LM-PMMA@Ilex chinensis Sims, @Photinia serrulata, and @Lotus. The I-V curves together with these from original cells, covered with LM-PMMA@corn were depicted in Fig. 3(b). Power increases were also observed. Noticing similar transparencies of the engineered polymers, we studied light harvesting efficiency influenced by the transmission haze rates. The efficiency increments at the maximum powers were plotted as a function of haze rate in Fig. 3(c). Generally speaking, photovoltaic efficiency increases with the haze rate. The PV tests were also performed on a photocell on a new PV unit. Similar increase trend was found after folding the LM-PMMA@corn on the cell (see Supplementary Information). Obvious increases of photocell powers were observed after putting the LM-PMMAs onto the photocells, despite of the PV cell stages.

## Discussions

Understanding the lightwave guidance mechanism in the biomemetic polymers will prompt designs and optimizations of textured structures. For this purpose, we dig out the origins of aforementioned optical properties, and discuss possible design routes to tailor light guiding films in light harvesting systems. While the high optical transparences are easily attributed to the PMMA’s intrinsic optical transparency, the origin of high haze values and low reflection needs elaborated experimental determinations and theoretical simulations. In the present work, the only difference during preparations is the selection of leaf species with different surface morphologies. Thus, we experimentally investigated the surface morphologies, and employed them as inputs in the Monte-Carlo simulations of light transport.

**Figure 4:**
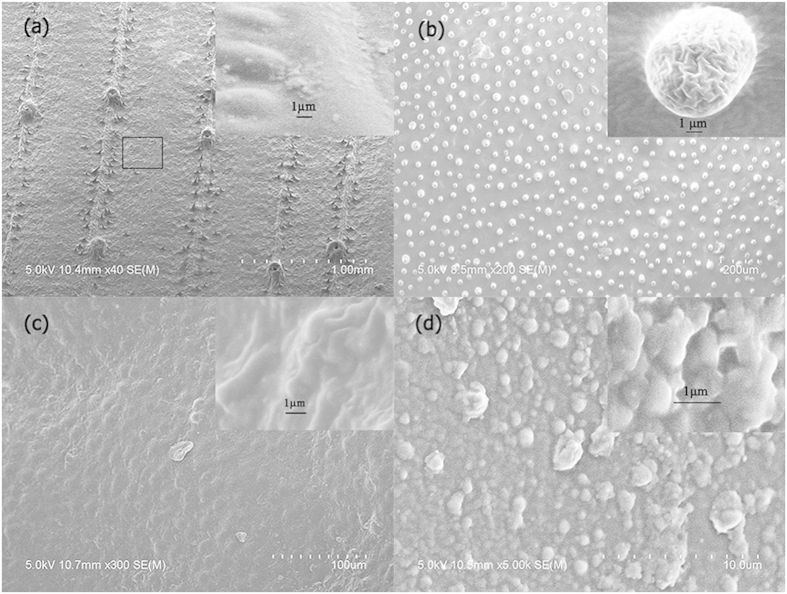
The scanning electron microscope images of leaf-mimicking poly-(methyl methacrylate) polymers. Master leaf species of are corn in (**a**), Lotus in (**b**), Photinia serrulata in (**c**), and Ilex chinensis Sims in (**d**). Onset show zoomed features in sub- micrometer scales.

The textured structures of the LM-PMMA polymers are mainly inherited from the master foliage. Various types of surface morphologies were found through scanning electron microscopy (SEM), and the roughness through atomic force microscopy (AFM). We selected the most typical films representing surface structures of corns, lotus, Photinia serrulata and Ilex chinensis Sims leaves. The SEM images were depicted in Fig. 4 (a–d), with the zoomed features in the onsets. More details of the morphologies and nanostructures of the same species can be found in the Supplementary Information. In general, the shapes are wrinkled in micrometer scales. The micro-wrinkles on the LM-PMMAs enlarge functional areas of light acceptances compared to flat PMMA or photocell surfaces. This suggests one of the origins of light harvesting efficiency increase.

We further studied differences of the surface morphologies of the LM-PMMAs. As shown in Fig. 4(a), the mimicked corn PMMA has regular stripe patterns, hemispherical dots, and trichomes. In Fig. 4(b), microstructures of LM-PMMA@lotus can be simply branched to smooth surface and papillae dispersed on the film. Such structures on the hard PMMA film are comparable to the lotus topographies casted on soft poly(dimethylsiloxane), and very similar to the lotus leaf structures^23^. In Fig. 4(c) imitating Photinia serrulata structures give raised wrinkles, 4-sided truncated pyramids together with hemispherical structures. The pyramids are clearer in AFM images of Supplementary Figure 4. This natural pyramid type coincides to anti-reflective structures etched on silicon surface^24^. The artificial Ilex chinensis Sims leaf has rather coarse surfaces consisting of hemispherical bubbles in sub-micrometer sizes. Inherited from master leaves, microstructures can further be decorated with nanostructures. These are found in the zoomed nano-dot and nano-bar features at onsets in Fig. 4 and Supplementary Information. The densities of micro- grains or strips also depend on plant species. Spacing among these structures is noticed in Fig. 4. However, a young leaf structure is not just a miniature of the one from the corresponding mature leaf. Supplementary Figure 7 shows the morphologies of LM-PMMAs from the same master plants as in Fig. 4. Clear similarities of main microstructures were found between polymers of young and mature master leaves. Differences are obvious in the structures such as papillae and stomata. Young lotus leaf replica image has more intensive papillae, yielding an increase of haze rate as shown in Supplementary Information. Indeed leaf structures changes not only among species but also during the leaf developments^25^,^26^,^27^, and among venation networks^28^. Study on leaf evolution and development is a dedicated subject^29^, and out of the present work’s scope. In what follows, we will focus on the optical properties due to the microstructures inherited from mature leaves, though these morphologies have other functions such as superhydrophobic media of lotus leaves^23^,^30^ and in helping being evergreen in winter of the ilex^31^.

**Figure 5:**
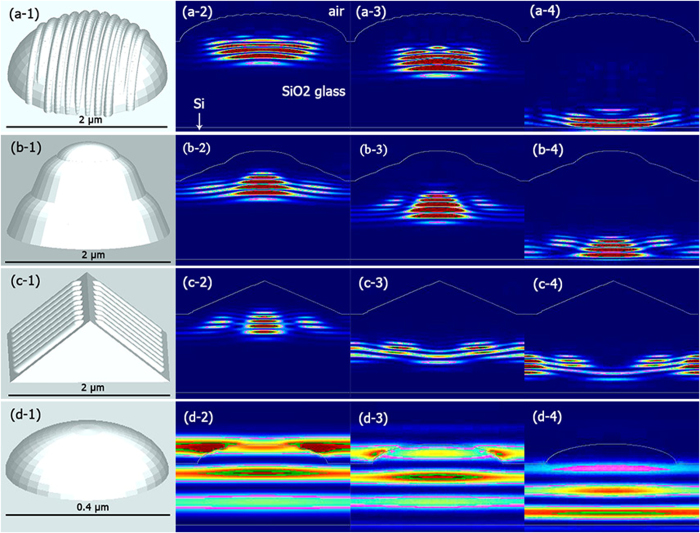
Monte-Carlo simulations of light transport. (**a**) A structure to mimic micro-grains on corn leaf surface consists of micro-hemisphere and nano-bars decorating the hemisphere. (**b**) Structure mimicking lotus surface grains has the cone structure in 3 stages. (**c**) 2 μm tetrahedron decorated with nanobars was shown to emulate the Photinia serrulata leaf surface. (**d**) A hemisphere with 0.4 μm was applied to approach the artificial Ilex chinensis Sims micro-morphologies. Photon propagations were shot at light entrance, interference, and about to exit films.

The textures on leaves are rather complicated due to their adaptations to various environments and functionalities. Based on microstructures determined through SEM and AFM, the morphologies were abstracted into regular models of nano-bar textured hemispheres (corn), round tip cones (lotus), 4-sides truncated pyramid structures (serrulata), and sub-micro hemispheres (ilex). In addition, the nano-hemispheric and bar structures were also added to the micro hemispheres, and 4-sides of pyramids. Diagrams of these structures were depicted in Fig. 5(a–d). Light propagations inside of the diagrams were carried out by means of Monte-Carlo simulations of photon transport^32^. Photon propagations and scattering dynamics are filmed in animations at Supplementary Movies. Snap shots of photon paths just after entering the structures, within the films, and about to exit the films were depicted for the modelled structures in Fig. 5(a–d). In general, photons are scattered and interfered inside of the multilayer structures. Directions of incident parallel photon beams are clearly diverged after the abstracted structures. Larger densities in unit area of the same diagrams will result in a larger divergence of parallel beam. This is corresponding to the high haze rates of the LM-PMMAs listed in Table 1. The divergent paths after the first 2–3 μm of light convergences make the photon diffuse inside of the polymers, resulting in large scattering areas as shown in Fig. 2. Size differences of monochromic lights after passing through the biomimetic polymers can be explained through classical optics mechanism where photons of smaller wavelengths will be less diverged by particles within present experimental conditions. The path of interferences and photon divergences are clearly seen in the figures and more vivid in animations.

**Figure 6:**
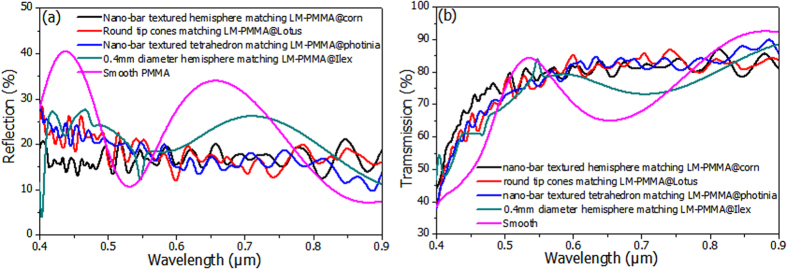
Calculated results of optical properties. In **(a),** optical reflection curves simulated from different abstracted structures are depicted. And **(b)** gives transmission curves influenced by the structures abstracted from LM-PMMA surface morphologies.

Introducing the nano-bars onto the micro-structures substantially decreases the reflection rates. Though the gratings of micro pyramid or hemisphere structures were reported to lower light reflections^33^, Rayleigh scatterings will further decrease the reflection when photons interact with the nanostrctures^34^. Reflection and transmission curves of the photons were depicted in Fig. 6(a,b). The nano-bar textured micro-hemispheres give the smallest reflections in all the regions down to 15%. Similar low reflection rate was also found for the nano-bar decorated truncated structure. These values are much less than the corresponding microstructures without nano features. The trend of nano-bar textured hemisphere <cone<tetrahedron (pyramid) <sub-micro hemisphere <glass are found in the reflection curve at Fig. 6(a). Meanwhile, rather high transmissions can be seen in Fig. 6(b). At this photon energy range of solar radiances, the photoabsorption in PMMAs or glasses are rather small, compared to the reflection and transmission. It is noteworthy that exact quantifications of haze rate to surface microstructure require elaborated works. Despite of counting the density effects of microstructures, veins and mesophylls on leaves should also be taken into considerations. Light scatterings and interferences through leaf structures in different height at wrinkles make simulations even more complicated. However, the present simulations employed main features as the texture serve direct supports to our experimental results of optical transmission and haze rates. In addition, presences of micro- and nano- textures lead to decreases of reflection and increases of photon travel path, and are attributed to another origin of light harvesting efficiency increases. The photon transport simulation was further extended to thicker models. Slab thicknesses were increased to 5 μm and 10 μm, while abstracted surface structures kept unchanged (see Supplementary Information). The reflection curves were found to have negligible changes. Transmissions were slightly decreased due to increases of light absorptions in thicker media, in accordance with the thickness dependent result in the previous section.

In conclusion, we engineered PMMA thin films to mimic leaf surface structures. The biomimetic polymers have high transparencies and transmission haze rates. Covering the films onto solar cells substantially increases photovoltaic efficiencies. The light harvesting magic of these LM-PMMAs in photon guidance is attributed to the combination of surface wrinkles and optical property changes lead by micro- and nano- structures. Both of the 3D wrinkles and tiny structures are inherited from their master leaf species. The transmission haze rates are further qualitatively explicated via Monte-Carlo simulations of light transport. We illustrated that micro- hemispheres decorated with nano-bars give best performances of anti-reflection. Bio-mimicking leaf surface structures may supply a strategic route in optimization of covers employed in existing photovoltaics. Learning from Mother Nature, we can imitate leaves from the natural plant library of more than 350000 species, and tailor the artificial film optical properties with careful selections of their mimicked masters. Present leaf-mimicking route can be employed as a cheaper and easier alternative in finding artificial micro-scale textures. The biomimetic films can be applied directly onto existing PV systems without reorganizing the rest of system structures. Beside potential applications to alleviate fossil fuel depletion, the foils imprinted with leaf can also be used in other optics such as mosaic roof in greenhouses for agriculture.

## Methods

### Experiments

Optical properties of the LM-PMMAs were measured through a WGT-S Transmission and Haze Rate Equipment (Shanghai Dianguang Electronics). A halogen lamp (DC 12V, 50W) was equipped and employed as the incident source. The operation scheme was in the integrating-sphere mode^35^. The instrumental precision is 0.01%, much smaller than statistic errors listed in Table 1. In the scattering measurements, a distance of 32 cm was selected between the laser source and projection screen. A digital camera was employed to record scattered features of the laser beams. Photovoltaic experiments were carried on commercially available crystalline Si solar cell units. Cells with different qualities were chosen, considering the real cases of the PV applications. The used one has a size of 5.45 cm × 5.45 cm, while the new one of 14.5 cm × 14.5 cm, much larger than most LM-PMMAs. The cell surfaces were directly folded with the LM-PMMAs covering the whole area of the small cell or partial of the big one. A 300 W Xenon lamp was chosen as the light source due to its spectral similarity to the sunlight^36^. The small PV cell was placed ~2 m from the artificial source. Light scattering and PV tests were performed in a dark room. At least 3 times were repeated for each sequence of measurements, by horizontal rotations or translations of the polymers on cells. In surface structure measurements, an S-4800 scanning electron microscopy was used to investigate the surface morphologies of the leaf-mimicking PMMAs. The three-dimensional surface morphology and the surface roughness were further characterized by AFM (CSPM4000). Polymers were coated with carbon if needed.

### Simulation

Based on the finite differential time domain (FDTD) solutions, Monte-Carlo simulations were carried out on the abstracted polymer surface structures with the 0.05 μm Si substrates through the software of FDTD solutions^TM^. The SiO_2_ glass was employed in the simulation instead of PMMA polymers due to the similarities of their optical parameters^37^ and photovoltaic experiment setups. The glass thicknesses were fixed to 3 μm, 5 μm and 10 μm. Though much less than the polymer thicknesses, the incident light propagations were mostly defined as found in the simulation at this distance. A modelling case was explicated in details in the Supplementary Information. As discussed in Discussion Section, we simplified the PMMA grain structures to nano-bar textured micro-hemispheres structures, cone, tetrahedron, and nano-hemisphere.

## Additional Information

**How to cite this article**: Huang, Z. *et al.* Replication of Leaf Surface Structures for Light Harvesting. *Sci. Rep.* **5**, 14281; doi: 10.1038/srep14281 (2015).

## Supplementary Material

Supplementary Information

Supplementary Movie 1

Supplementary Movie 2

Supplementary Movie 3

Supplementary Movie 4

## Figures and Tables

**Table 1:** Transparencies and haze rates of leaf-mimicking poly-(methyl methacrylates) with mimicked master leaf names.

Plant Names	Transparency(%)	Optical haze (%)	Plant Names	Transparency(%)	Optical haze (%)
Ligustrum vicaryi (0.59 mm)	83.8 (1.0)	87.6 (0.8)	Weigela florida (0.58 mm)	85.1 (2.7)	65.7 (1.1)
Corn (0.55 mm)	84.1 (1.5)	84.5 (1.7)	Rosa chinensis Jacq. (0.63 mm)	86.5 (1.8)	64.7 (0.9)
Pittoaporum tobira (0.63 mm)	85.2 (1.6)	83.4 (1.0)	Sapium sebiferum (0.62 mm)	86.5 (1.8)	64.7 (0.7)
Sugarcane (0.65 mm)	76.8 (1.3)	82.3 (0.6)	Euonymus alatus (0.6 mm)	87.8 (0.9)	63.8 (1.1)
Prunus salicina Lindl (0.57 mm)	87.0 (2.7)	81.0 (0.9)	Pear (0.59 mm)	86.2 (2.4)	63.5 (2.1)
Phoenix Tree (0.61 mm)	81.9 (1.3)	80.5 (1.6)	Kerria japonica (0.57 mm)	86.2 (1.3)	63.5 (1.7)
Camphor (0.63 mm)	82.9 (1.1)	77.6 (0.7)	Michelia figo (0.58 mm)	87.4 (0.8)	62.0 (1.1)
Red Loropetalum chinense (0.58 mm)	85.6 (0.9)	76.3 (1.3)	Lotus Magnolia (0.64 mm)	85.6 (2.4)	61.9 (1.9)
Ginkgo biloba L. (0.56 mm)	83.8 (0.7)	76.0 (0.7)	Rosa banksiae (0.63 mm)	87.8 (0.7)	59.4 (0.9)
Lotus (0.63 mm)	85.1 (2.4)	75.7 (0.9)	Scholar tree (0.61 mm)	87.6 (1.9)	58.4 (2.1)
Osmanthus fragrans (0.59 mm)	85.6 (2.6)	74.8 (3.7)	Parthenocissus (0.57 mm)	85.6 (4.6)	57.4 (1.7)
Brunfelsia latifolia Benth (0.55 mm)	84.6 (1.8)	74.1 (0.9)	Ilex chinensis Sims (0.56 mm)	87.7 (1.6)	54.2 (2.2)
Euonymus fortuneivar (0.62 mm)	81.8 (1.1)	73.5 (0.6)	Buxus megistophylla (0.60 mm)	87.9 (0.8)	51.6 (0.7)
Ficus elastica (0.57 mm)	78.3 (2.0)	69.2 (1.6)	Berberis thunbergii (0.58 mm)	88.7 (1.8)	48.9 (1.2)
Photinia serrulata (0.61 mm)	85.9 (0.3)	68.3 (0.5)	Nerium indicum (0.57 mm)	91.5 (0.3)	26.7 (1.4)
Nandina domestica (0.60 mm)	85.0 (3.0)	66.0 (1.5)	Smooth PMMAs (0.62 mm)	90.9 (0.6)	5.2 (1.0)

Average biomimetic film thicknesses were tabulated after each plant name in the parenthesis. Typical thickness error is ± 0.05 mm. Standard deviations of the transparency and haze rates are in the parenthesis.
