# Supplementary material for: Replication of Leaf Surface Structures for Light Harvesting
Source: Sci Rep. 2015 Sep 18;5:14281. doi: 10.1038/srep14281 (PMC4585631; doi:10.1038/srep14281)
Supplement: Supplementary Information [file srep14281-s1.doc]

Supplementary Information for Replication of Leaf Surface Structures for Light Harvesting

Zhongjia Huang ^1 ,*^, Sai Yang^1^, Hui Zhang^2^, Meng Zhang^3,*^, and Wei Cao^4^

^1^School of Mechanical and Automotive Engineering, Anhui Polytechnic University, Wuhu 241000, China

^2^Soochow University-Western University Centre for Synchrotron Radiation Research, Institute of Functional Nano and Soft Material (FUNSOM) and Collaborative Innovation Center of Suzhou Nano Science & Technology, Soochow University, Suzhou 215123, China

^3^Department of Physics, East China University of Science and Technology, Shanghai 200237, China

^4^ Research Center for Molecular Materials, University of Oulu, P.O. Box 3000, FIN-90014, Finland

^*^ These authors are contributed equally to this work.

Correspondence and requests for materials should be addressed to Z.H (email: [hzj@ahpu.edu.cn](mailto:hzj@ahpu.edu.cn) ) or to W. C. (email: [wei.cao@oulu.fi](mailto:wei.cao@oulu.fi)).

The thickness dependent experiment was carried out by varying the LM-PMMA@corn thicknesses. The optical transparency and haze rate were depicted in Supplementary Figure 1.

**Supplementary Figure 1|** Transparency and haze of the LM-PMMA@corn as a function of the film thickness.

The optical transparency and haze of the biomimetic PMMA may be influenced by the structures of master leaves during leaf developments. Young leaves of corn, lotus, Photinia serrulata, and Ilex chinensis Sims were employed as the masters. Together with film thicknesses, visible light transparencies and the haze rates of the corresponding PMMA were tabulated in Supplementary Table 1.

**Supplementary Table 1|** Transparencies and haze rates of leaf-mimicking poly-(methyl methacrylates) with mimicked master leaf names. Young leaves were employed in the polymer engineerings. Average biomimetic film thicknesses were tabulated after each plant name in the parenthesis. Typical thickness error is ±0.05 mm. Statistical errors of the transparency and haze rates are in the parenthesis.

| Plant Names | Transparency (%) | Optical haze (%) |
| --- | --- | --- |
| Corn (0.62 mm) | 87.4 (0.6) | 81.8 (2.1) |
| Lotus (0.59 mm) | 86.7 (0.5) | 85.4 (1.9) |
| Photinia serrulata (0.61 mm) | 86.8 (0.3) | 65.4 (2.2) |
| Ilex chinensis Sims (0.62 mm) | 87.8 (0.6) | 55.6 (1.3) |

Supplementary Table 2 shows parameters of different photocells employed in the PV measurements. Errors raised during experiments were negligible provided the same experimental conditions.

**Supplementary Table 2|** Parameters of photovoltaic cells employed in the present study. Sizes were in cm, while nominal and measured open circuit voltages in volt.

| PV cell | Size (cm) | Nominal U_oc_ (V) | Measured U_oc_ (V) |
| --- | --- | --- | --- |
| Used One | 5.45 × 5.45 | 3 | 2.91 (0.01) |
| New One | 14.50 × 14.50 | 7.2 | 7.20 (0.01) |

The I-V curves of a used Si cell were shown in the main content to simulate real cases of the PV applications. It has been noticed that the cell has a relatively low fill factor (FF) of ~0.4. Impacts on the FF may arise from many causes. In the present case, the relatively low FF was rather from the performance losses of the photovoltaic with the using time. During usages, more extrinsic defects would appear, resulting in surface recombination loss in the Si-based PV cells^1^. Moreover, increases of the internal series resistance with time would bring in additional losses in energy yield^2^. Another sequence of solar light harvesting measurement was carried out. The I-V curve and I-P curves of photovoltaic cell covered by the artificial corn leaf were measured through a solar module analyzer (PROVA-200, TES Electrical Electronic Co.) under the sunshine radiation in 23^rd^ September 2014 at Wuhu, China. The weather was sun shining with light haze and low humidity. Due to relatively weaker solar radiance compared to the xenon lamp in lab, a larger photovoltaic cell with 14.5×14.5 cm^2^ was employed. It was partially covered by the artificial corn leaf of 5×5 cm^2^. The I-V and I-P curves were depicted in Supplementary Figure 2 (a) and (b) respectively. An obvious increase of photocell powers was detected too.


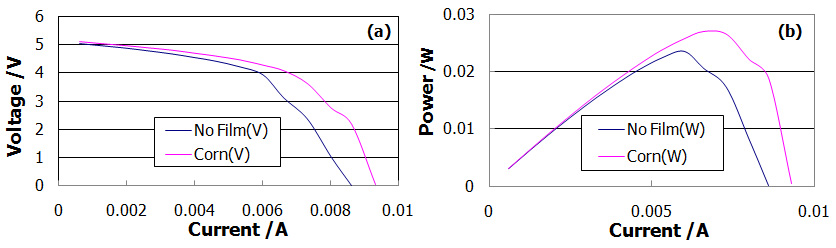


**Supplementary Figure 2|** (a) I-V and (b) I-P curves of a photo cell covered with an artificial corn leaf under the sunshine measured through the solar module analyzer.

Supplementary Figures 3-6 show more details of surface structures of LM-PMMA@corn, lotus, photinia serrulata, and ilex chinensis sims, respectively. In each sequence, the SEM images were depicted in (a)-(d), while AFM images at (e)-(f) for 3D morphological determination.


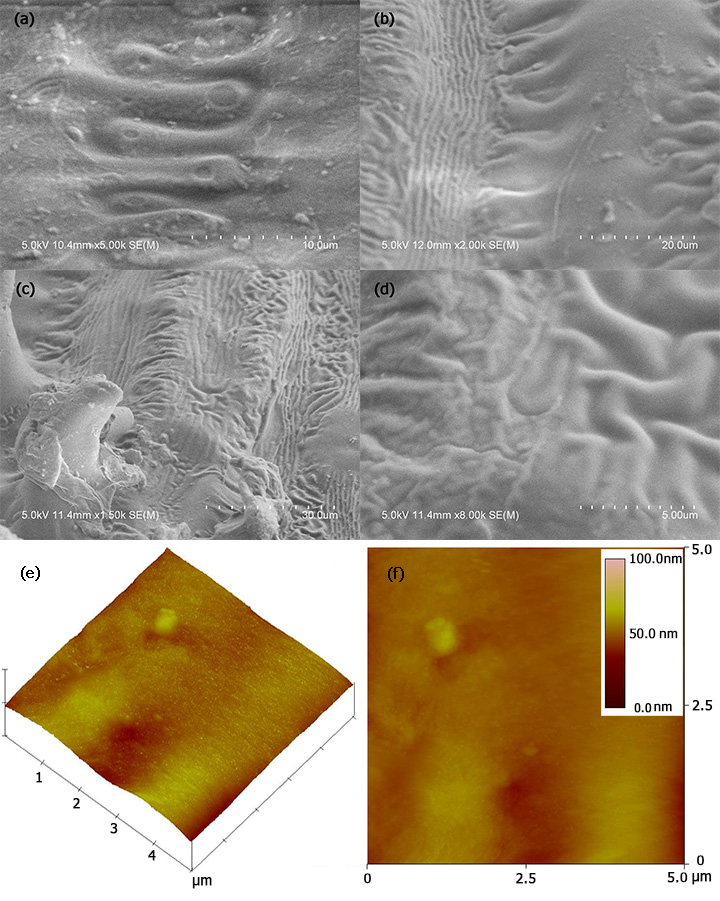
**Supplementary Figure 3|** Micro morphological structures of the LM-PMMA@Corn measured through scanning electron microscope in different magnifications at (a)-(d), and atomic force microscope at (e)-(f).


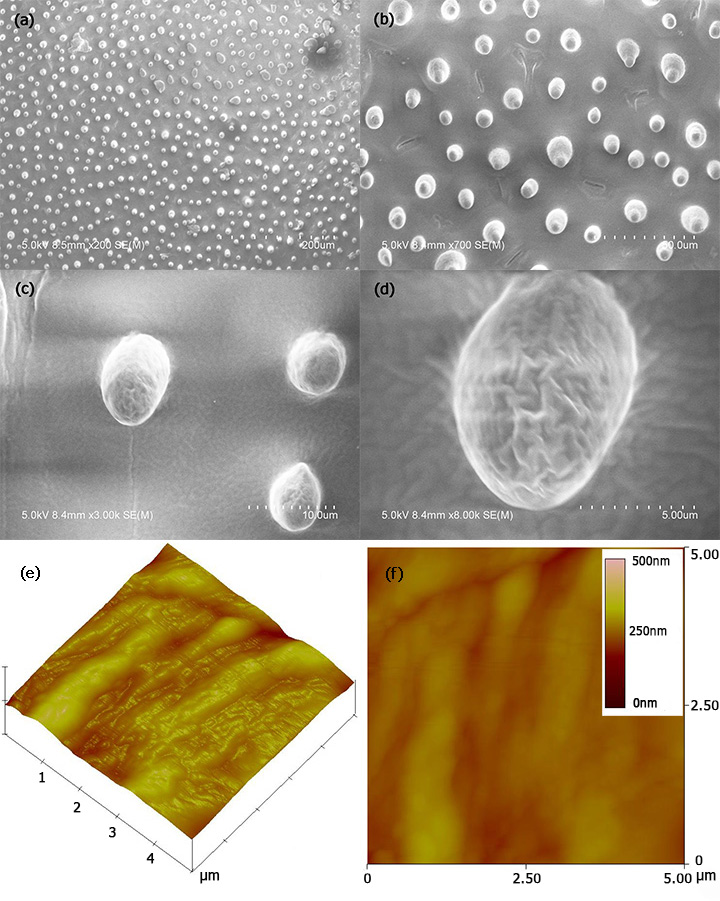
**Supplementary Figure 4|** Micro morphological structures of the LM-PMMA@lotus measured through scanning electron microscope in different magnifications at (a)-(d), and atomic force microscope at (e)-(f).


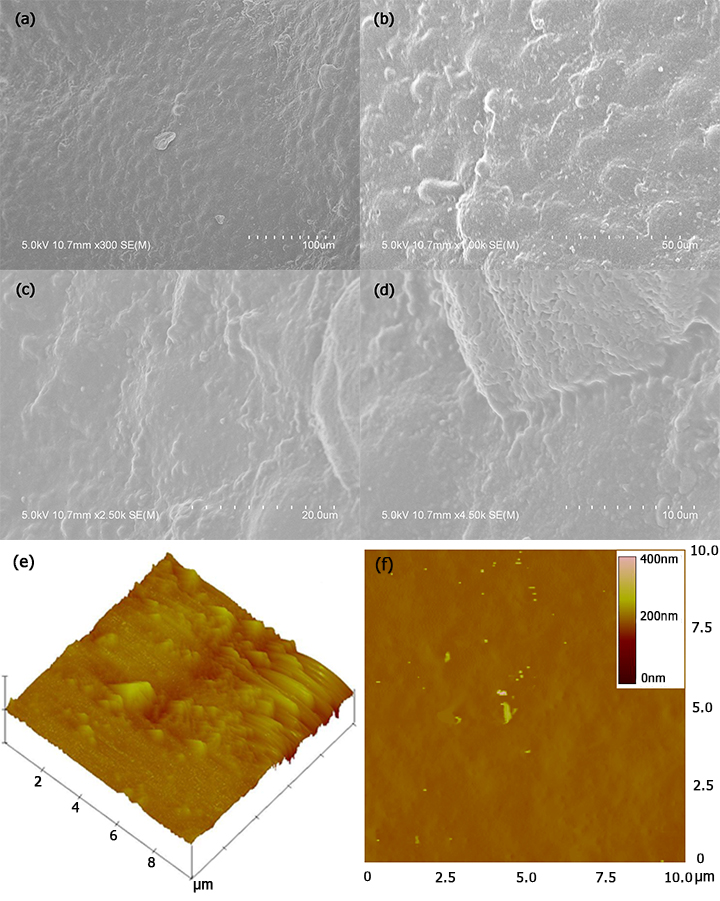
**Supplementary Figure 5|** Micro morphological structures of the LM-PMMA@photinia serrulata measured through scanning electron microscope in different magnifications at (a)-(d), and atomic force microscope at (e)-(f).


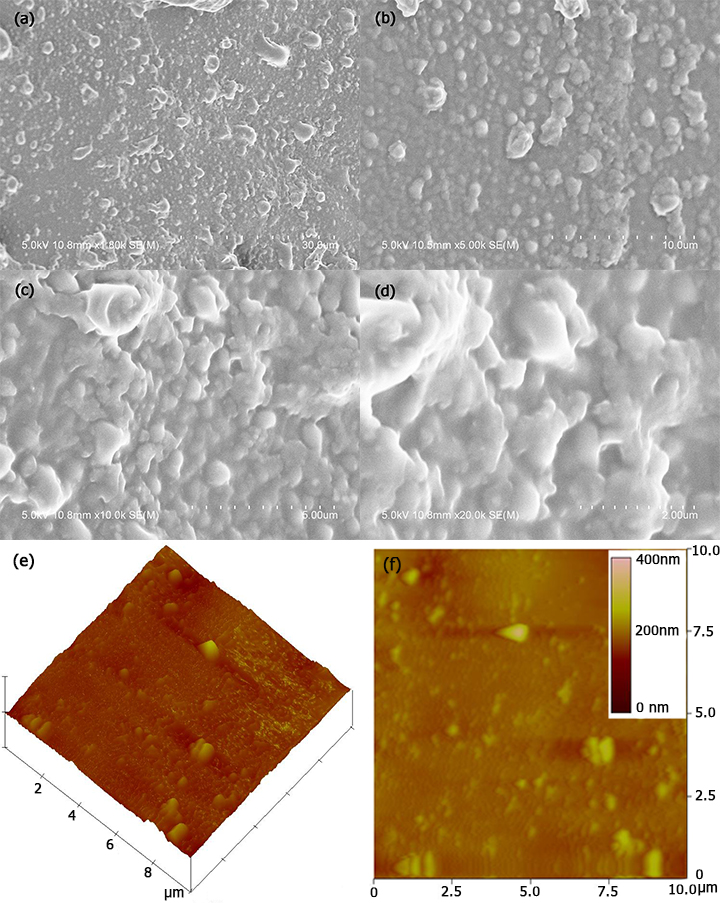
**Supplementary Figure 6|** Micro morphological structures of the LM-PMMA@ ilex chinensis sims measured through scanning electron microscope in different magnifications at (a)-(d), and atomic force microscope at (e)-(f).

Supplementary Figure 7 shows the surface structures of LM-PMMA@corn, lotus, photinia serrulata, and ilex chinensis sims, respectively. Young leaves of the plants were employed as the masters during the polymer engineering.


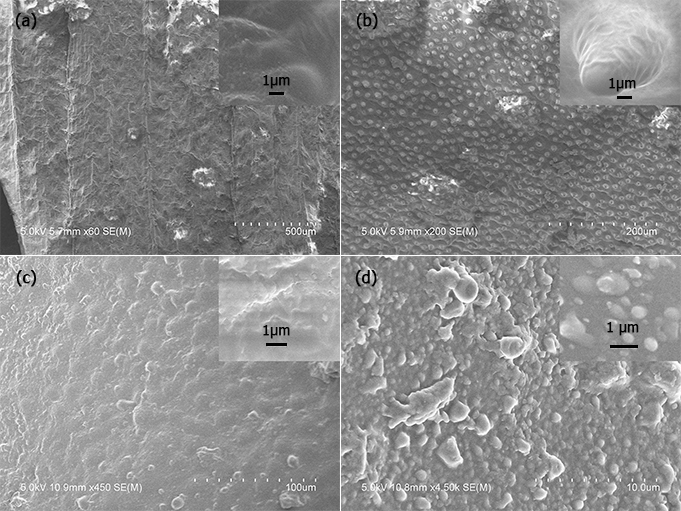


**Supplementary Figure 7|** Scanning electron microscope images of leaf-mimicking poly-(methyl methacrylate) polymers. Master young leaf species are from corn in (a), Lotus in (b), Photinia serrulata in (c), and Ilex chinensis Sims in (d). Onset show zoomed features in sub- micrometer scales.

Monte-Carlo simulations were also carried out by varying the SiO_2_ thicknesses. The results were given in the form of light reflection and transmission in Supplementary Figure 8 for the abstracted structures of the LM-PMMA@corn. No big changes were found either in the reflection of transmission as a result of thickness changes. Similar works were also performed for another 3 structures generated from the present LM-PMMA@ lotus, Photinia serrulata, and Ilex chinensis Sims. The reflection and transmission curves were depicted in Supplementary Figures 9 with the oxide slab thickness of 5 µm, and 10 of 10 µm.

**Supplementary Figure 8|** Reflection (a) and transmission (b) curves calculated via Monte-Carlo simulation of light transport for nano-bar textured hemisphere matching LM-PMMA@corn with different SiO_2_ slab thicknesses.

**Supplementary Figure 9|** Reflection (a) and transmission (b) curves calculated via Monte-Carlo simulation of light transport for abstracted structures employed from LM-PMMA@ corn, lotus, Photinia serrulata, and Ilex chinensis Sims with SiO_2_ slab thicknesses of 5 µm.

**Supplementary Figure 10|** Reflection (a) and transmission (b) curves calculated via Monte-Carlo simulation of light transport for abstracted structures employed from LM-PMMA@ corn, lotus, Photinia serrulata, and Ilex chinensis Sims with SiO_2_ slab thicknesses of 10 µm.

**Supplementary Methods: Simulation.**

Light transportations inside of the mimicked films were simulated through the Monte-Carlo method by using the commercial available software of FDTD Solutions, Lumerical Solutions Inc. Key methodology is based on the discretization of the time-dependent Maxwell's equations (in partial differential form) to the space and time partial derivatives. Central-difference approximation was employed as the numerical algorithm. Modellings were carried out by taking menus of Structures, Components, Simulation, Sources, and Monitors. More details of the software, instructions, operations, and methodology can be found e.g., within the software Reference Guide v. 6.5.

A schematic drawing of the FDTD simulation is shown in Supplementary Figure 11. In the present study, physical models were set by taking the following materials and parameters from the bottom to the top. A 0.05 µm Si slab was placed at the bottom as the photovoltaic semiconductor. It was folded with SiO_2_ layers varied in thicknesses to imitate the covers. Above the oxide layers, micro-structures decorated with nano- grains or –strips were introduced in the same SiO_2_ material. Taking the abstracted structure of the LM-PMMA@corn as an example, we have shaped the micro-structure to a 2 µm hemisphere, on whose surface toroid bars of 100 nm diameter are evenly spaced. Margins in the horizontal plane were fixed more than 0.1 um, and periodically repeated. In the vertical direction, boundary conditions were selected to the perfectly matched layers. In the simulation, plane wave with the wavelength from 400 nm to 900 nm were chosen from the software as the radiation source. Animations were filmed to monitor the photon pass, and frequency domain filed and power to the reflection and transmission.


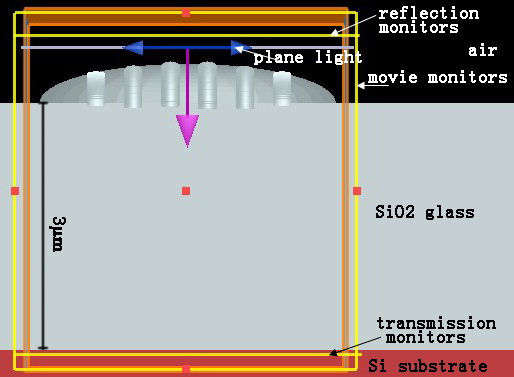


**Supplementary Figure 11| Abstracted model of the LM-PMMA@corn employed in Monte-Carlo simulations.** An example of the model includes surface structures, protection glass, and Si substrate in photovoltaic cells. Reflection monitor and transmission monitor were set above the system, and below the SiO_2_ slab.

**References**

1. Aberle, A. G. Surface Passivation of Crystalline Silicon Solar Cells: A Review. Prog. Photovolt: Res. Appl. 8, 473-487 (2000).

2. Kunz, G. & Wagner, A. Internal Series Resistance Determination of Only IV-Curve under Illumination. 19th Eur. Photovolt. Solar Energ. Conf., 5BV.2.70 (2004).
